# Protein Hydrolysis by Subcritical Water: A New Perspective on Obtaining Bioactive Peptides

**DOI:** 10.3390/molecules26216655

**Published:** 2021-11-03

**Authors:** Carlos I. Rivas-Vela, Silvia L. Amaya-Llano, Eduardo Castaño-Tostado, Gustavo A. Castillo-Herrera

**Affiliations:** 1 Departamento de Investigación y Posgrado en Alimentos, Facultad de Química, Universidad Autónoma de Querétaro, Querétaro 76010, Mexico; carl.rivas.mx@gmail.com (C.I.R.-V.); samayal@uaq.mx (S.L.A.-L.); ecastano@uaq.mx (E.C.-T.); 2 Unidad de Tecnología Alimentaria, Centro de Investigación y Asistencia en Tecnología y Diseño del Estado de Jalisco, A.C., Camino Arenero # 1227, Col. El Bajío Arenal, Zapopán 45019, Mexico

**Keywords:** subcritical water hydrolysis, bioactive peptide, protein

## Abstract

The importance of bioactive peptides lies in their diverse applications in the pharmaceutical and food industries. In addition, they have been projected as allies in the control and prevention of certain diseases due to their associated antioxidant, antihypertensive, or hypoglycemic activities, just to mention a few. Obtaining these peptides has been performed traditionally by fermentation processes or enzymatic hydrolysis. In recent years, the use of supercritical fluid technology, specifically subcritical water (SW), has been positioned as an efficient and sustainable alternative to obtain peptides from various protein sources. This review presents and discusses updated research reports on the use of subcritical water to obtain bioactive peptides, its hydrolysis mechanism, and the experimental designs used for the study of effects from factors involved in the hydrolysis process. The aim was to promote obtaining peptides by green technology and to clarify perspectives that still need to be explored in the use of subcritical water in protein hydrolysis.

## 1. Introduction

Research on food proteins has recently increased due to properties beyond their nutritional function. Protein research has been focused on techno-functional characteristics and on multiple biological activities to discover new bioactivities in humans that may contribute to preventing or treating diseases. Much of the scientific interest is directed toward finding and evaluating bioactive peptide sequences that can reduce or prevent the risk of noncommunicable diseases, such as diabetes and hypertension [1]. A bioactive peptide (BAP) is defined as a sequence of 2–20 amino acids, with a molecular weight less than 3 kDa, encoded within a parental protein that, when released, presents a specific activity with a beneficial impact on human health. The biological activities of the peptides are closely related to their release of amino acids by a hydrolysis process that imparts a free active form that could exert biological activities [2]. Different studies have reported peptide bioactivities such as antioxidant, anticarcinogenic, antihypertensive, antimicrobial, anti-inflammatory, antithrombotic, antidiabetic, mineral-binding activities, antimicrobial, dipeptidyl–peptidase IV-inhibitory, opioid, and immunomodulatory activities that have been shown in in vitro and in vivo studies [3,4,5,6].

Bioactive peptide release can occur through different processes, by enzymes in the gastrointestinal tract or by food processing [7] or endogenous peptides released by biochemical pathways necessary for metabolic processes within organisms such as carnosine or glucagon [8]. Different processing methods have been developed for releasing peptides from their parental proteins: enzymatic hydrolysis (using digestive enzymes or proteolytic enzymes from plants or microbes) or a fermentation process [6].

In the case of enzymatic hydrolysis, trypsin and pepsin, both digestive enzymes, are widely used in obtaining peptides with different activities, such as antioxidant or angiotensin-converting enzyme (ACE) inhibitory activity [9,10]; the use of plant enzymes such as papain and bromelain has also been reported [11]. The combination of different enzymes can be used to obtain bioactive peptides [12].

Although the use of enzymes has a specificity at an amino-terminal, which allows for predicting which peptides can be generated, it is a high-cost process, requiring long periods of time, and uses acids and bases to control the pH process, generating polluting effluents; in addition, in some cases, salts derived from the neutralization of solutions add extra steps for peptide separation or purification [13]. On the other hand, the use of a fermentative process to obtain bioactive peptides from food proteins, in general, has the advantage of being performed in food matrices ready-to-eat, with the benefits of the biological activities not only from the released peptides but other benefits from the matrix itself (such as the presence of prebiotics and probiotics). However, fermentations have certain limitations as they are less controllable and more variable, take a longer time, and are less scalable [14]. Some examples of the microorganisms used are lactic acid bacteria such as *Lactobacillus plantarum* [15] and molds such as *Aspergillus oryzae* [16]. Likewise, obtaining bioactive peptides has been performed in some instances by employing acids and alkalis; however, compared to the aforementioned methods, these have less specificity and control, generating effluents that affect the environment [13].

In this context, the use of subcritical water hydrolysis (SWH) for the release of bioactive peptides of different food proteins has gained attention as a powerful green technology for the extraction of compounds or the hydrolysis of different polymers to its monomers [17]. Subcritical water (SW) is also called hot-compressed water or pressurized hot water, because it is defined as water under a range of pressures and temperatures, above its boiling temperature at 1 atm (>100 °C at 0.1 MPa) and below its critical point, with a pressure sufficient to maintain its liquid state (374.5 °C at 22.06 MPa) [18]. Under these conditions, the physicochemical properties of the water change significantly in dielectric constant (ε), viscosity, or ionic product constant (Kw). At room temperature (25 °C), its ion product constant (K_w_) is 10^−14^ mol^2^ L^−2^, but as the temperature increases, K_w_ increases by three orders of magnitude, reaching 10^−11^ mol^2^ L^−2^ at 300 °C. However, above this temperature and as it approaches its critical point, K_w_ decreases abruptly to 10^−20^ mol^2^ at 380 °C. The increase in K_w_ values is due to a combination of oxygen–hydrogen stretching within each molecule and vibrations between molecules by the effect of the rising temperature, resulting in the formation of hydroxide (OH^−^) and hydronium (H_3_O^+^) ions in the aqueous medium; the increase in these ions increases the reactivity of the water, allowing it to act as an acid, base, or double catalyst for hydrolysis reactions [19,20]. With the loss of hydrogen bonds in the water molecules due to the change in temperature in the subcritical region, the values of the dielectric constant (ε) of the water begin to decrease, behaving increasingly as a nonpolar solvent. At room temperature, ε is 80 and has the capacity to dissolve or interact with polar or ionic compounds; at 225 °C, it decreases to ε = 30, adopting properties of other solvents such as methanol and finally descending to ε = 6 just below the critical point (374.5 °C), with properties similar to those of nonpolar solvents such as hexane [21].

These particular properties of the water are due to its high hydrogen-bonding capacity, reflecting changes in viscosity, superficial tension, diffusivity, or high boiling point.

The main applications of subcritical water have been in the extraction of bioactive compounds from many food matrixes, such as flavonoids from mandarin [22] or black tea [23], anthocyanins [24], pectin [25], gallotannins [26], polyphenols from different fruits [27], caffeine [28], and essentials oils [29,30]. Likewise, its application in the hydrolysis of biopolymers such as polysaccharides and proteins has already been explored in coconut meal [31], monosaccharides for the production of ethanol from sugarcane bagasse [32], and in the release of amino acids from different sources, such as de-oiled rice bran [33], rapeseed cake [34], raw soybean [35], onion waste [36], bean dregs [37], scallop viscera entrails [38], or poultry intestines [39].

**Figure 1:**
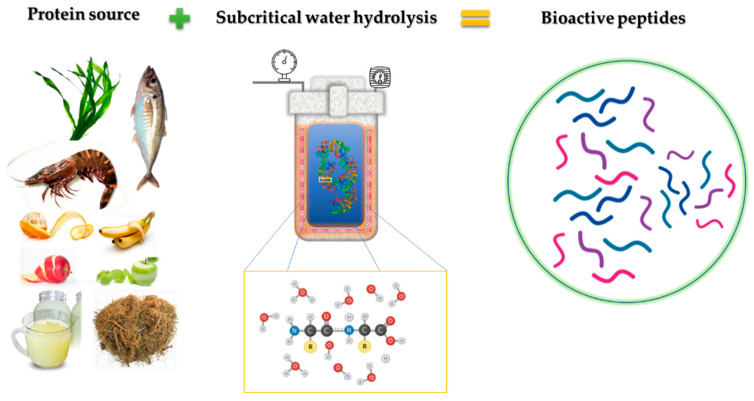
SWH general scheme.

For a long time, the application of SW in proteins has focused mainly on obtaining amino acids but not on their intermediates, the peptides. However, in recent years, research has focused on peptide release as an alternative method to enzymatic hydrolysis. In this sense, the main objective of the present paper was to provide a review of the use of subcritical water hydrolysis (SWH) as a powerful tool for obtaining bioactive peptides (Figure 1).

## 2. Subcritical Water Hydrolysis

### 2.1. Sources

#### 2.1.1. Vegetable Proteins

The application of subcritical water as a hydrolysis medium has been explored in different protein sources, both in vegetal and animal proteins, as well as other sources. Watchararuji and coworkers [35] studied the application of subcritical water in two agro-industrial byproducts: rice bran and soybean meal. The hydrolysates with the best antioxidant activities (measured by ABTS) were obtained at 220 °C after 30 min of reaction in both materials; however, the highest recovery of peptides took place at 220 °C in the rice bran hydrolysates and at 200 °C for the soybean meal. In other research, Sereewatthanawut’s team [33] evaluated the effects of an SWH process to de-oiled rice (*Oyaza sativa*) bran to obtain hydrolysates with functional activities and to obtain a value-added product from this byproduct. The results showed that the best condition to promote the antioxidant activity of hydrolysates was at 200 °C for 30 min, without significantly affecting the preservation of amino acids.

#### 2.1.2. Animal Proteins

The use of subcritical water as a medium of protein hydrolysis has been explored mainly in proteins of animal sources, mainly from byproducts derived from fish industries, although its application is not limited only to these. The conditions of the processes to this type of protein source are variable; depending on the objective and the protein origin, temperatures ranging from 170 °C to 250 °C have been used, as well as reaction times from five to ten minutes up to 6 h, highlighting both variables as being correlated with each other, as when using high temperatures, the periods are a few minutes, while longer periods are used at lower temperatures. In addition to this, when compared with hydrolysates from other protein sources to obtain bioactive peptides with animal proteins, temperatures and times were used at higher levels, and hydrolysates with multiple biological activities were obtained.

During food processing, different byproducts are generated, and the fish industry has some byproducts with interesting protein content, one of them being tuna skin. Ahmed and Chun [40] applied SWH in tuna skin from Bigeye tuna, and collagen was extracted by supercritical CO^2^. The best treatment for the release of bioactive peptides was at 280 °C for 5 min for both materials; however, the best degree of hydrolysis was identified at 250 °C. The obtained hydrolysates showed antioxidant and antimicrobial activities. The antioxidant activity was evaluated by ABTS, DPPH, FRAP, and metal-chelating activity; these activities were compared with hydrolysates obtained by enzymes in previous studies, demonstrated as higher than this last one. The antimicrobial activity was effective against three microorganisms of interest for the food industry: *Staphylococcus aureus*, *Pseudomonas aeruginosa*, and *Bacillus cereus,* which demonstrated the potential of this hydrolysate for different purposes.

Another example of the application of SWH in fish proteins has been reported by Hao’s team [41]; in this case, they used abalone (*Haliotis discus hannai* Ino) viscera as a protein source with the aim of adding value to this byproduct by obtaining hydrolysates with antioxidant activities. The SW was used as a one-step extraction and hydrolysis process, consisting of the application of a temperature range from 110 °C to 230 °C for one hour and sufficient pressure to maintain the liquid state, finding the best response at 170 °C with yield extractions of 46% and 60.85% protein concentrations; the molecular distribution of the hydrolysate ranged mainly from 1000 to 5000 Da (41%), and the highest antioxidant activity was by DPPH, hydroxyl radical scavenging activity, reducing power, and lipid peroxidation inhibition.

Álvarez and coworkers [42] studied the effect of SWH coupled to oxygen injection in porcine hemoglobin, a byproduct of the meat industry with potential as a functional ingredient. The hydrolysate with the best characteristics was generated under treatment at 180 °C, 0.4 MPa for 240 min, with a release of 83% of peptides with low molecular weight (2.1 kDa), a reduction in their color, an enhancement in their antioxidant properties, and good functional properties (solubility and emulsifying) when compared to native hemoglobin or that obtained by enzymatic hydrolysis.

Enteshari and Martínez-Monteagudo [43] studied the effect of SWH on the protein fraction of ice-cream wastewater for obtaining bioactive peptides and adding value to this byproduct under process conditions of temperatures of 130–230 °C and pressures of 2–6 MPa, using a continuous stirred-tank reactor. The maximum value of the degree of hydrolysis reached 41% at a temperature of 230 °C for 240 min and an interesting part of the reported results was the analysis of the experimental data of the degree of hydrolysis and predicted values by the Weibull distribution model, with good correlation *R*^2^ = 0.981, which enabled a kinetic model and apparent activation energy of the hydrolysis by SW of 37.53 ± 5.21 kJ mol^−1^. The hydrolysates obtained presented good bioactivity as an antioxidant and ACE-inhibitory activity with a maximum inhibition (98.0 ± 1.6%) at 230 °C.

Cho’s group [44] used subcritical water in a shrimp (*Penaeus japonicus*) powder used as a food ingredient. In the different treatments, they identified that the hydrolysate generated at 200 °C and 3 MPa for 10 min exhibited greater antioxidant activity and had better physicochemical characteristics. The hydrolysate showed a strong free-radical scavenging capacity evaluated by DPPH, ABTS, and FRAP (3.08 ± 0.04, 26.74 ± 0.06, and 12.12 ± 0.50 mg TEAC/g dried mass, respectively).

The giant African snail *Achatina fulica* is considered in some areas as a pest that puts different crops at risk; with the available information on its composition, alternatives have been sought for its use. Due to its high protein content (62.89%), Cho et al. [45] evaluated the effect of SW at different temperatures, from 100 °C to 300 °C for 10 min, over different characteristics such as physicochemical and bioactive activities such as ACE inhibitory, acetylcholinesterase inhibitory, and antioxidant activities by DPPH, ABTS, and FRAP methods. The results obtained by the authors indicate different conditions depending on the product or biological activity that is the objective, offering different possibilities for the same material by SWH: the antioxidant activities were highest in the hydrolysate at 250 °C; ACE inhibitory activity at 200 °C; and acetylcholinesterase inhibitory activity at 300 °C. However, for a hydrolysate that presented “balanced” bioactivities, the best condition was 250 °C, 2.5 MPa, for 10 min.

#### 2.1.3. Other Sources

The valuing of other alternative protein sources has also been explored through the application of SW, as in the case of some algae, with the intention of obtaining or enhancing food functionality or bioactive peptides for pharmaceutical or food purposes.

Park’s team [46] applied an SWH experimental design to the laver *Pyropia yezoensis* to obtain hydrolysates with antioxidant activities and to improve its functionality as a food ingredient. The treatments consisted of the exploration of different temperatures from 120 to 230 °C, 3 MPa for 30 min with constant stirring at 200 rpm. The antioxidant activities were evaluated by DPPH, and the ABTS of the hydrolysates obtained was higher than those obtained by two control treatments, identifying the optimal treatment at 210 °C with a radical-scavenging activity of 16.63 ± 0.20 for DPPH and 19.45 ± 0.07 for ABTS, both in milligrams of Trolox per gram of dried mass.

Ultrasound coupled to a subcritical water process is an innovative approach for the release of bioactive peptides that was recently reported. Fan’s group [47] designed and developed a piece of equipment coupled to the ultrasound system inside the reactor to evaluate its effect on obtaining BAPs from *Spirulina platensis*, an algae with a high protein content (50–70%). The results under this new system were more efficient in all senses (time, yields, and molecular distribution) compared to an SW hydrolysis process using a reactor without any modification. With the data retrieved and using the response surface methodology, the optimal treatment for peptide release could be predicted (153 °C, 221 W, 64 min, and 10 MPa), and the molecular distribution of the hydrolysate generated by ultrasound treatment was compared with those generated by the application of five proteases: papain, pepsin, trypsin, Alcalase, and Protamex. The ultrasound procedure yielded principally small molecular peptides (<1000) (57.48%), while the enzyme processes showed mainly peptides between 1 and 5 kDa, demonstrating the effectiveness of the new method for the release of lower-molecular-weight peptides without using enzymes or processes at high temperatures. The same research team reported the application of this new method to obtain antidiabetic peptides, evaluating its effectiveness in the inhibition of three relevant enzymes: α-amylase, α-glucosidase, and dipeptidyl peptidase-4 (DPP-IV) and later in an insulin-resistant HepG2 cell model. The best inhibition activity in the three enzymes was obtained by treatment at 120 °C, 10 MPa, an ultrasound power of 220 W for 60 min, without showing a cytotoxic effect in the HepG2 cells, significantly increased glycogen content, hexokinase, and pyruvate kinase activities compared with the treatment control, and finally concluding with the identification of 11 peptides found in the hydrolysate, and selecting and evaluating the bioactivity of only three (GVPMPNK, RNPFVFAPTLLTVAAR, and LRSELAAWSR) [48].

The application of SW to obtain bioactive peptides, as already mentioned, has been shown to have applications mainly in protein sources of animal origin; however, due to its characteristics, it has also shown to be an adaptable technology to different protein sources and even flexible for the modulation of the hydrolysis process focused on a specific activity or on the balance of biologic activities that can be obtained.

### 2.2. Parameters and Operation Conditions

The best conditions and bioactivities of the different hydrolysates obtained by SWH reported are summarized in Table 1. In general, for animal source protein, hydrolysis requires higher temperatures or a longer processing time compared to vegetable proteins, as well as those of algae, the latter two sources presenting very similar conditions, as observed in the case of the laver *Pyropia yezoensis*, soybean, and rice bran. As can be seen, some of the sources from which bioactive peptides have been obtained have been byproducts of the food industry or materials already classified as waste, and as mentioned by Marcet’s team [13], these materials are sometimes rich in proteins with the possibility of being used to obtain different biomolecules with great potential, demonstrated by the generation of bioactive peptides. In addition, the possibility of scaling up and then reusing the water used in the procedures, similar to what has been seen when using supercritical CO_2_, and the coupling of different technologies, such as ultrasound, add some advantages to the use of this technology compared to conventional hydrolysis processes, which has promoted the exploration of the specific obtaining of peptides from more traditional protein sources [49].

Information from reports of protein hydrolysis by subcritical water showed some interesting characteristics. Initially, correlations between process times and temperature are easily appreciated, as for a longer reaction time, a lower temperature is applied and vice versa. It is understood that the increase in temperature promotes an increase in the hydrolysis rate constants and, with it, a reduction in the reaction time. Attention to the conditions of the process is relevant as the objective is to obtain bioactive peptides, because, although the increase in temperature reduces the process times, accelerating the process can lead to a greater release of amino acids by the medium, promoting the degradation of larger amino acids (more thermosensitive) into smaller amino acids [51], and increasing the products of the Maillard reaction (increasing the browning) [55] or the generation of products derived from the hydrolysis of the latter degradation products such as organic acids [56]. The kinetics of the reactions that can be carried out during an SW process are discussed in the next section.

## 3. Hydrolysis Mechanism

**Figure 2:**
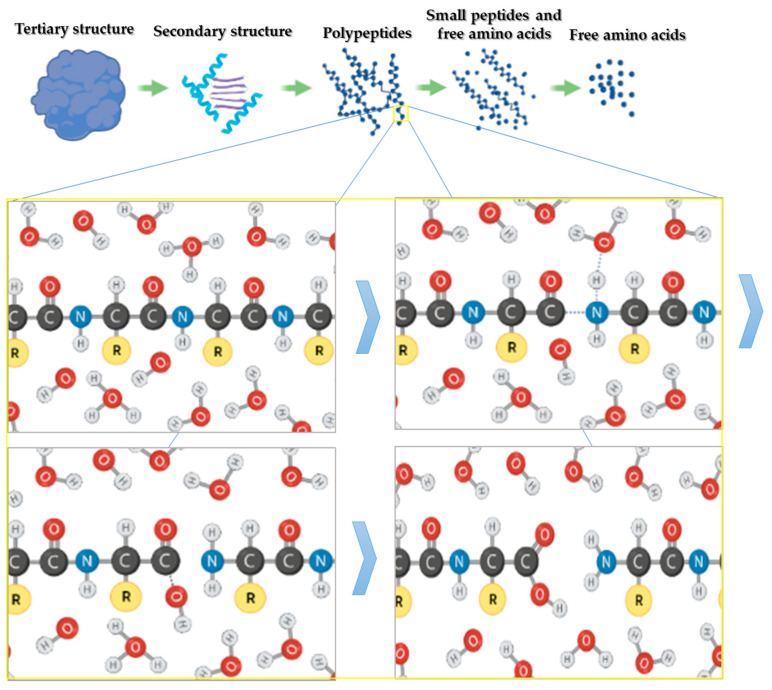
Scheme of protein hydrolysis mechanism of subcritical water.

The effect of high pressure and high temperature separately has not shown to be effective for the hydrolysis of proteins to obtain small peptides and amino acids, as their effect is limited to the denaturation of proteins, that is, losses of quarter, tertiary, and even the secondary structures [56,57]. It is only under the joint effects of high pressure and temperature that the conditions for hydrolysis of the peptide bond between amino acids occurs [58]. The mechanism of hydrolysis of the peptide bond has been assumed, in general, under an irreversible first-order reaction model, identifying as the catalysts of the reaction to hydronium and hydroxyl ions present in the medium, conferring bicatalytic characteristics to water, although it has also been assumed to be a weak acid or base [59]. The stages of the protein hydrolysis process are shown in Figure 2, wherein in the beginning, high pressure and temperature promote the disruption of weak interactions, such as hydrogen bonds, producing the loss of quaternary (if present), tertiary, and secondary structures [49,57,60]. In the peptide bond (see Figure 2), first, the union of a proton H^+^ (derived from the hydronium ion) to the N-terminal generates atom excitation that leads to the cleavage of the peptide bond, and then the ion OH- joins to the new carbon cation of the C-terminal [61]. The reaction due to the bicatalytic characteristics of water could be carried out in the same way with the union at the onset of the ion OH^−^ and after the proton H^+^. It is relevant to mention that the increase in ions modifies the pH of the medium correlated with the temperature of the treatment, observing a tendency to decrease at temperatures below 180 ° C and increase above 200 °C, mainly associated with the degradation of amino acids and reaction products of Maillard [45,51,62].

Espinoza’s team [63] mentioned that the selectivity by which subcritical water hydrolyzes peptide bonds is not exactly clear, so it is difficult to conclude which peptide bonds are more susceptible to hydrolysis. In addition, it is known that, during the overheating process, water molecules tend to form clusters. Although, with the available information, it is difficult to explain the interactions in smaller clusters, the cage effect, or the solute–solvent collisions, it seems that many of the reaction sites are governed by randomness, which makes their prediction difficult. If the conditions are known, the method could allow for increasing the concentration of desired amino acid residue in the peptides. Nonetheless, an important advance for the understanding of the SWH mechanism has been presented by Powell and coworkers [18], evaluating its effect on three reference proteins (hemoglobin, bovine serum albumin, and β-casein) to deepen the selectivity of the SW cutting for peptide release. With the results obtained from treatments at different temperatures (160, 207, 253, and 300 °C) and up to 20 min treatment, they showed that, although the residue cut pattern may be different depending on the protein evaluated, it is possible to observe a trend in amino acid residues such as aspartic acid (mainly in treatments at 160 °C) and glutamic acid, associating this trend with a weak acid hydrolysis mechanism. In addition to these findings, some limitations of the split of the disulfide bonds present in bovine serum albumin were observed. Something else to take into account is that in SW, treatment has the possibility of generating modifications in the side chains of some amino acids, such as the oxidation of cysteine, methionine, and tryptophan [64].

## 4. Design of Experiments

The statistical theory of experimental design can provide advantages such as the efficient identification of significant main and interaction effects using a few runs. It is considered a systematic approach for answering experimental questions and correctly analyzing the generated data [65].

Experimental designs used in the research of supercritical fluid technology application processes, associated with their intrinsic characteristics, have followed two approaches, exploratory experiments and optimization research [66]. The choice of experimental design will depend on the type of research or available information before experimenting. In exploratory research, the so-called screening designs are used for discrimination among many factors; for those with the greatest effects on the response variable of interest, designs such as the series of full factorial, 2^k^, and their fractions are used in this type of research. On the other hand, optimization designs are implemented once there is sufficient information regarding the influencing factors by trying to find optimal factor levels for the response, designs such as the Box–Behnken and Central Composite designs [66], or the usage of the so-called alphabetically optimal designs [67].

As mentioned previously, the use of 2^k^ factorial designs is for exploratory purposes, where a factorial design intends to delve into two selected levels of each factor, at the extremes of the experimental zone intended to be analyzed (represented as low level: −1, and high: +1). This is an efficient strategy to obtain valuable information in a few runs with a large number of factors to be distinguished [68]. The factorial design is an excellent approach in the early stages of experimentation or when there are a relatively large number of factors to be explored in SWH research. In this sense, Fan and coworkers [47] used a factorial design for exploration purposes where an ultrasound probe (as a new factor) was coupled to the SW reactor to obtain peptides from Spirulina platensis. The factors of temperature, pressure, time, and ultrasound power were explored. With the results obtained, they were able to identify most important main and interaction effects and then discard one of the factors (pressure) and leave it at a fixed value in a subsequent optimization phase. However, the most widely used approach in SW protein hydrolysis processes has been through unifactorial design (OFAAT: One Factor At A Time), exploring the effects of temperature and treatment time, in turn, given that the effect of pressure on the response has been observed as a nonsignificant effect. In OFAAT, effects from temperature are explored over a wide range of values, normally between 100 and 300 °C, keeping time and pressure fixed [15,36,39,41,49]. Hao and coworkers [41] used a unifactorial approach to evaluate the effect of temperature from 110 °C to 230 °C in the antioxidant activities and protein hydrolysis of abalone viscera. Similarly, Melgosa and coworkers [51] used a temperature range up to 250 °C to hydrolyze sardine protein to improve its antioxidant and antiproliferative biological activity. It is always important to keep in mind, nonetheless, that when there would be interaction effects among factors, OFAAT experimentation is inefficient in the usage of resources and potentially induces a loss of information when more factors than temperature and pressure are involved in the process or some component within the raw material may have an effect on the performance of the same process [69]. In contrast, factorial designs do allow the identification and analysis of interactions between factors. Currently, our ongoing research on the use of subcritical water as a proteolytic medium and adding modifiers has given us information on the dependency of effects that exist between the concentration of the modifier, temperature, and pressure on the response variables, information that we could not obtain if the approach of the experimental design were not factorial.

Optimization designs are commonly made after exploratory experiments to locate an experimental region with a few factors and to estimate quadratic effects, toward the identification of optimal conditions. The number of reports available for the implementation of optimization designs using SWH is limited. An example of a Box–Behnken design was also used by Fan and coworkers [47], evaluating the three factors selected for this stage: temperature, time, and power of an ultrasound treatment; with the factor in three levels and with the data derived from the experimental runs of this design, they were able to identify the optimal empirical conditions for the release of peptides, subsequently corroborating the identified optimal conditions with an error rate of 0.17%. Another example of the implementation of optimization designs is given by Espinoza’s team [70], after the use of a fractional factorial design 2^3^, they used a rotatable central composite design having as the response variable the degree of hydrolysis in a whey protein isolate, varying the temperature of the process and the time processing and concentration of the modifier used. With the results presented by a response surface graph, it was possible to identify a treatment that reached a degree of hydrolysis around 50%.

### 4.1. Subcritical Water Factors

#### 4.1.1. Temperature

SW hydrolysis is primarily governed by temperature, inducing irreversible reactions of the first order as already mentioned; the increase in temperature produces changes in the physicochemical properties of water such as its diffusivity, surface tension, energy, or dielectric constant. In addition, the increase in the process temperature accelerates hydrolysis, consequently opening possibilities of other process times [35]. Taking into account that hydrolysis requires different conditions depending on the source of protein (vegetable or animal) [29,48], the range of temperature will be a function that depends on the source protein. Finally, hydrolysis conditions will depend on the type of bioactivity of the products [45].

#### 4.1.2. Pressure

Pressure is a key factor for keeping the water below the vapor phase and, therefore, in its liquid phase at high temperatures, allowing a subcritical state. Although different pressure values have been explored from the minimum above the vapor pressure to 10 MPa, unlike the temperature, pressure modulation in OFAAT designs does not show a significant effect on the mechanism or performance of hydrolysis; however, its effect can be significant when more factors are taken into account, such as 2^k^ factorial approaches [69]. Likewise, the choice of the gas that generates the pressure in the equipment has proven to be a factor to take into account, in equipment where pressurization is generated by the presence of these [71].

#### 4.1.3. Hydrolysis Time

The time of the process depends primarily on the product to be obtained and, in turn, on the temperature range at which it is intended to work; high temperatures will require less time and vice versa. The study of a hydrolysis process can be performed by a static perspective (time fixed) or from a kinetic viewpoint, and then correspondingly to evaluate effects from different temperatures and other factors, and/or their interactions [72]. A kinetic approach aims to deepen the process dynamics, to understand the time trend of the stability or conversion rate of the amino acid profile of the hydrolysate [56,73].

#### 4.1.4. Gas Atmosphere of Reactor

Pressure is generated by the insertion of a specific gas or by a dry air compressor. The selection of the type of gas to be inserted in the SW-generating process has proven to be important, mainly in the extraction phase. The possibilities are nitrogen, carbon dioxide, and oxygen. In searching for reductions in reaction times or specific modifications in amino acid profiles or the produced peptides, gases are also considered catalyzers [71].

#### 4.1.5. Modifiers

The use of catalysts or modifiers has been shown to have the potential to modulate hydrolysis alongside reducing reaction times or minimizing the process temperature to obtain the desired product. Some of the additives or compounds explored have been acetic acid, carbon dioxide, oxygen, oleic acid, lactic acid, sodium hydroxide, sodium bicarbonate, hydrochloric acid, sulfuric acid, and sodium chloride (See Table 2). Espinoza and Morawicki [70] explored the effect of five modifiers (acetic acid, lactic acid, sodium chloride, and sodium bicarbonate) on time, temperature, and degree of hydrolysis in whey. Sodium bicarbonate increased the degree of hydrolysis four times and the production of free amino acids up to 44%, and it decreased the molecular weight of the produced peptides. Moreover, a positive effect was observed in the conservation of some amino acids such as arginine and histidine. Improvement of the protein hydrolysis by modifiers was previously reported by Kang and Chun [74] in silk fiber, when they evaluated four modifiers at different concentrations: formic acid, salt (NaCl), oleic acid, and NaOH; they observed that the addition of 5 mol% of formic acid and NaOH significantly increased the hydrolysis yield by three and four times, respectively.

A gas not only acts as a pressure generator but also as a modifier. Marcet and coworkers [71] evaluated the effect of pure nitrogen and oxygen atmospheres on the hydrolysis of an extract of insoluble egg proteins. The results concluded that compared to the nitrogen treatment, peptides with decreased functional properties were obtained when oxygen was used; this may be due to the fact that, derived from the use of oxygen, structural changes may occur in the amino acids of the recovered peptides. Zhong’s group [76] observed an increase in the hydrolysis rate of whey under a CO_2_-only atmosphere, attributing its effect to the promotion of catalysis by a carbonic acid derivate in the presence of CO_2_.

The use of modifiers has shown that it can negatively and positively influence the preservation of amino acids, modifying the final amino acid profile of the hydrolysates, so it is essential to evaluate this effect in specific protein sources and research objectives [70].

#### 4.1.6. Material to Be Hydrolyzed

It is important to take into account the characterization and preparation of the material prior to being treated in order to achieve the desired hydrolysates, such as the particle size, the product of origin, and its composition, as the presence of other compounds could affect the performance of the hydrolysis process [77]. Even though there are no concise reports on the effect of decomposition products of food matrix compounds on biological activities or process yields, for example, materials with considerable amounts of fat (>10%) are generally defatted, mainly using a supercritical CO_2_ method [40,51,55], prior to the treatments to avoid its probable influence in the process [78].

#### 4.1.7. Other Variables

Solute and solvent ratios, protein source, or reactor type are variables that, although they have shown a lower impact on the hydrolysis process, it is important not to leave them aside, because they do have an impact on hydrolysis yields. An example of this is the importance of the solute-to-solvent ratio and the particle size, as the greater the surface exposed to subcritical water, the better the mass flow, which can improve the efficiency of the hydrolysis process [79].

## 5. Conclusions

Recent applications of subcritical water as a medium for the release of bioactive peptides have confirmed it as a new powerful green alternative tool for this purpose. The characteristics of this technology allow effective modulation of the process parameters, for a specific response and, in parallel, allow flexibility to incorporate different adaptations to the same process such as the use of different gases, modifiers, or coupled technologies, such as ultrasound.

Although, until now, protein cleavage specificity and its control or prediction are not entirely clear, when SW hydrolysis is compared with enzymatic hydrolysis, it represents a flexible controllable process. Moreover, recent reports have enabled the elucidation of some characteristics of the mechanisms in some reference proteins; using this valuable information together with an adequate experimental design, it should be possible to obtain SW hydrolysates from different protein sources in an efficient way.

Then, the substitution of chemical or fermentive hydrolysis by faster and more ecological and efficient processes such as SW hydrolysis will be possible; although the initial acquisition cost of supercritical fluid equipment is currently high, it is important to evaluate its benefits and long-term advantages, and it is possible to scale the process up at the pilot or industrial level, which have already been carried out in laboratory processes.

Further research is required to verify the SW mechanisms. Moreover, its effect on diverse protein sources should be studied, as well as its modulation and comparison with others already implemented in the agro-food industry. Likewise, more research is required on the possible effects of the peptides or hydrolysates obtained by this method on human nutrition and health, as the modifications to the side chains and other modifications in the amino acid profile have not been evaluated to rule out health risks using a clean technology.

## Figures and Tables

**Table 1:** Bioactivities and operation parameters found in the bibliography of different protein sources.

Scheme	Sample Proportion and Protein Concentration	Bioactivity	Type of Reactor	Optimal Conditions	References
Tuna (*Thunnus obesus*) skin	Skin: 3 g (no data), Collagen extract: 0.75 g (no data), 150 mL of water	Antioxidant and antimicrobial	Closed	280 °C, 8 MPa, 150 rpm, 5 min	[40]
Porcine hemoglobin	20 g (no data), 400 mL of water	Antioxidant	Closed coupled to oxygen injection	180 °C, 4 MPa, 360 min	[42]
Mackerel skin and bones	1 g (86.89–90.05%), 200 mL of water	Antioxidant	Closed	250 °C, 7 MPa, 150 rpm, 3 min	[50]
Sardine (*Sardina pilchardus*)	60 g (52.2–66.2%), Water (no data)	Antioxidant and antiproliferative	Closed	250 °C, 10 MPa, 30 min	[51]
Laver (*Pyropia yezoensis*)	35 g (35.21%), 665 mL of water	Antioxidant	Closed	210 °C, 3 MPa, 200 rpm, 30 min	[46]
Soybean and rice bran	Rice bran: 1.76 g (15.02%), Soybean meal: 1.76 g (43.55%), 7 mL of water	Antioxidant	Closed	200 °C, 3.97 MPa, 20 min	[35]
Tuna collagen	0.75 g (no data), 150 mL of water	Antioxidant and antimicrobial	Closed	250 °C, 5 MPa, 0.6 M of modifier, 3 min	[52]
Giant African snail *(Achatina fulica)*	3.2 g (no data), 160 mL of water	ACE inhibitory, acetylcholinesterase (AChE) inhibitory, and antioxidant	Closed	Antioxidant: 200 °C ACE inhibitory: 250 °C AChE inhibitory: 300 °C 2.5 MPa, 10 min	[45]
Squid muscle	6 g (73.26%), 150 mL of water	Antioxidant	Closed	220 °C, 140 rpm, pressure of saturated steam, 3 min	[53]
Ice-cream wastewater	(6.81% dry-basis), 350 mL of ice-cream wastewater	Antioxidant and antihypertensive	Closed	230 °C, 6 MPa, 240 min	[43]
Algae *(Spirulina platensis)*	25 g (75.31%), 500 mL of water	Anti-diabetes	Closed coupled ultrasound	120 °C, 200 W, 10 MPa, 60 min	[48]
Abalone viscera	100 g (57–68.5%), 1500 mL of water	Antioxidant	Closed	170 °C, 0.8 MPa, 60 min	[41]
Oyster (*Crassostrea gigas*)	5.5 g (37.47%), 165 mL of water	Antioxidant and antihypertensive	Continuous	225 °C, 10 MPa, 150 rpm, 5 min	[54]
Blue mussel (*Mytilus edulis*)	30 g (52.87–67.30%), 600 mL of water	Antioxidant and antihypertensive	Closed	Antioxidant: 240 °C Antihypertensive: 180 °C, 3 MPa, 30 min	[55]

**Table 2:** Modifiers and their effects on protein hydrolysis by subcritical water.

Source	Catalyst/Modifiers Used	Optimal Conditions and Best Modifier	Main Effect	References
Whey protein	Acetic acid, lactic acid, sodium bicarbonate, sodium chloride, and sodium hydroxide	291 °C, saturated vapor pressure, 28 min, 1.24 M of sodium bicarbonate	Four-fold increase in the degree of hydrolysis compared to water alone. Positive conservation of tyrosine, serine, glutamic acid, glycine, isoleucine, methionine/valine, phenylalanine, and tryptophan.	[70]
Silk fibroin	Formic acid, sodium chloride, oleic acid, and sodium hydroxide	250 °C, 4 MPa, 32 min, 5 mol% sodium hydroxide	Four-fold increase in yield of amino acids.	[74]
Bovine serum albumin	Carbon dioxide	250 °C, 25 MPa, 300 s, 90% CO_2_ saturation	Increase in amino acid yield.	[58]
Chicken intestine	Sulfuric acid	260 °C, saturated vapor pressure, 28 min, 0.02 wt%	Increase in amino acids yield up to 11.49%.	[39]
De-oiled mackerel muscle	Nitrogen, carbon dioxide, formic acid, acetic acid, sodium chloride, and sodium bicarbonate	260 °C, 7 MPa, 3 min, 150 rpm, 0.6 M of sodium bicarbonate	Increase in hydrolysis yield. Higher antioxidant and ACE-inhibitory activities.	[75]
Insoluble Egg Yolk Granular Protein	Oxygen and nitrogen	180 °C, 4 MPa, 120 min, oxygen stream of 1 L/min	Up to two-fold increase in amino acid yield and reduced time compared to trypsin digestion.	[71]

## Data Availability

Not applicable.
